# New Drugs, Appliances, and Things Medical

**Published:** 1893-05-13

**Authors:** 


### NEW DRUGS, APPLIANCES, AND THINGS MEDICAL.

[All preparations, appliances, novelties etc., of which a notice is desired, should be sent for the Editor, to care of The Manager, 428, Strand, London, W.C.]

### MALTO-PEPSIN.

The earlier preparations of pepsin that proved so useful, although often so disagreeable, would no longer be appreciated after such an elegant and physiologically desirable preparation as Morse's Malto-pepsin. It is a potent digestive agent, and in some conditions it presents many advantages over simple preparations of pepsine. Its composition is stated to be: Saccharated pepsine, 10 grs.; saccharated pancreatine, 5 grs.; acid lacto-phosphate of lime, 5 grs.; exsiccated extract of malt, 10 grs., and thus it represents the digestive juices of the stomach pancreas and salivary glands. We have found it a very efficient and reliable preparation.

